# Glucagon-Like Peptide-1 Receptor Agonists, Semaglutide, and Atrial Fibrillation: An Umbrella Review With Methodological Appraisal of Overlapping Meta-Analyses

**DOI:** 10.7759/cureus.113641

**Published:** 2026-07-30

**Authors:** David Ramzy, Rouba Isshak, Disha Patel, Michael Elmasry, Noma Shri, Jesus Romero, Proggya Paromita, Tashifa Nazmee, Fayez Shamoon

**Affiliations:** 1 Internal Medicine, St. Joseph's Regional Medical Center, Paterson, USA; 2 Internal Medicine, St. Joseph’s Regional Medical Center, Paterson, USA; 3 Cardiology, St. Joseph's Regional Medical Center, Paterson, USA

**Keywords:** atrial fibrillation (af), glp-1 agonist, obesity treatment, semaglutide, umbrella review

## Abstract

Background: Multiple meta-analyses report that glucagon-like peptide-1 receptor agonists (GLP-1 RAs), and semaglutide in particular, are associated with lower incident atrial fibrillation (AF) and reduced post-ablation AF recurrence. These reviews have appeared in rapid succession, often drawing on the same trials.

Objective: To systematically identify, appraise, and reconcile the published meta-analyses examining GLP-1 RAs/semaglutide and AF across two clinical questions-incident AF and post-ablation AF recurrence.

Methods: We conducted an umbrella review following Preferred Reporting Items for Overviews of Reviews (PRIOR) reporting guidance. Eligible units were systematic reviews with meta-analysis reporting a pooled AF estimate for GLP-1 RA/semaglutide versus comparator. Each review was independently appraised in duplicate with A Measurement Tool to Assess Systematic Reviews 2 (AMSTAR-2); overlap of included primary studies was quantified with the corrected covered area (CCA) within clinical domains; certainty was summarized using a Grading of Recommendations Assessment, Development and Evaluation (GRADE)-informed framework. Between-review discordance was examined against pre-specified analytic features (comparator, statistical model, population, and pivotal-trial inclusion).

Results: Ten meta-analyses were included, comprising seven evaluating incident AF and three assessing post-ablation AF recurrence. One meta-analysis of incident AF was available only as a conference abstract and was reserved for sensitivity analysis. For incident AF, pooled effect estimates ranged from an odds ratio (OR) of 0.54 (95% confidence interval (CI) 0.39-0.77) to 0.83 (95% CI 0.70-0.98). For post-ablation recurrence, hazard ratios (HRs) ranged from 0.58 (95% CI 0.42-0.79) to 0.78 (95% CI 0.61-0.99). AMSTAR 2 ratings indicated low confidence in five reviews and critically low confidence in four; none achieved moderate or high confidence. Primary study overlap was substantial, with CCA values of 34.1% across the five semaglutide-only reviews of incident AF, 22.5% across all six incident AF reviews, and 43.8% across the three recurrence reviews. Two recurrence reviews shared an identical six-study base (pairwise CCA 100%) yet reported different pooled HRs (0.58 vs 0.78). The incident-AF effect gradient tracked comparator (active < placebo), statistical model (fixed < random), and population (type 2 diabetes < obesity).

Conclusions: The current evidence comprises numerous but low-confidence, substantially overlapping meta-analyses whose consistency partly reflects a shared set of primary trials rather than independent replication. GLP-1 RAs/semaglutide are plausibly associated with lower AF burden, but certainty is low (incident AF) to very low (recurrence). Adequately powered randomized trials with adjudicated, systematically monitored AF endpoints are required.

## Introduction

Glucagon-like peptide-1 receptor agonists (GLP-1 RAs), and semaglutide in particular, have rapidly become central to cardiometabolic care, with weight loss, glycemic, and cardiovascular benefits established across large randomized trials. A parallel hypothesis - that GLP-1 signaling favorably remodels the atrial substrate through reductions in epicardial adipose tissue, inflammation, fibrosis, and atrial stretch - has driven intense interest in atrial fibrillation (AF) as a potential additional beneficiary of these agents.

Two distinct clinical questions have been addressed. The first is whether GLP-1 RAs/semaglutide reduce incident (new-onset) AF, examined largely through pooled adverse-event data from weight management and cardiovascular outcome trials. The second is whether peri- or post-ablation GLP-1 RA therapy reduces AF recurrence after catheter ablation, examined through observational cohorts and a small number of randomized data.

Over approximately two years, both questions have generated multiple systematic reviews with meta-analyses reaching broadly concordant, favorable conclusions. This proliferation creates a recognized hazard in evidence synthesis: when many reviews draw on the same underlying studies, their agreement can be mistaken for independent replication, and methodological weakness can be masked by volume [1,2]. Umbrella review methodology addresses this situation by appraising the quality of the reviews themselves with AMSTAR-2 [1], quantifying the degree to which they share primary studies via the corrected covered area (CCA) [2], and examining discordance in terms of analytic choices rather than new data [3].

The distinction between apparent and actual replication is not merely semantic. Guideline developers and clinicians increasingly encounter dashboards in which a count of “positive” meta-analyses is treated as a proxy for strength of evidence. Where those meta-analyses recycle a common core of pivotal trials, the effective sample of independent evidence is far smaller than the number of syntheses implies, and their concordance provides limited additional certainty [2,3]. Calibrating this literature therefore requires explicit measurement of methodological quality [1], primary study overlap [2], and the analytic sources of any between-review disagreement.

We therefore conducted an umbrella review of meta-analyses of GLP-1 RAs and AF, spanning both incident AF and post-ablation recurrence. Semaglutide is named alongside the class throughout because most eligible reviews restricted their scope to semaglutide specifically, whereas a minority pooled the broader GLP-1 RA/co-agonist class; the two samples partly different trials, so the distinction is interpretive rather than redundant. The primary aim was to appraise the methodological quality (A Measurement Tool to Assess Systematic Reviews 2 (AMSTAR-2)), primary study overlap (CCA), and internal consistency of the existing synthesis literature. The secondary aim was to provide a calibrated, Grading of Recommendations Assessment, Development and Evaluation (GRADE)-informed summary of what is - and is not - established for each clinical question.

## Materials and methods

Protocol, reporting standards, and registration

This umbrella review was designed and reported in accordance with the Preferred Reporting Items for Overviews of Reviews (PRIOR) statement [3], and study identification and selection were reported in accordance with the Preferred Reporting Items for Systematic Reviews and Meta-Analyses (PRISMA) 2020 statement [4]. The review protocol was specified a priori. The review was not prospectively registered in the International Prospective Register of Systematic Reviews (PROSPERO). The project was initially conceived as a pairwise meta-analysis and was reframed as an umbrella review before the search was executed, at which point the a priori protocol was finalized; prospective registration was not completed at that stage, and we did not seek retrospective registration so as not to imply prospective status. Apart from this change of design, made before any data were extracted, no deviations from the a priori protocol occurred during the conduct of the review. To support transparency and reproducibility, the full a priori protocol is provided as Appendix A and will be deposited in a public repository upon acceptance; it also remains available from the corresponding author on request.

Eligibility criteria

We included published systematic reviews with meta-analysis that (i) evaluated a GLP-1 RA or semaglutide intervention; (ii) reported a quantitative pooled estimate (odds ratio (OR), risk ratio (RR), or hazard ratio (HR)) for AF as either an incident event or post-ablation recurrence; and (iii) compared the intervention against placebo, standard care, or an active comparator. We excluded narrative reviews, primary studies, editorials, and reviews not reporting a pooled AF estimate. Conference abstracts were eligible for identification but, because their abbreviated reporting precludes full critical appraisal, were not subjected to AMSTAR-2 and were reserved for sensitivity analysis. No language or date restrictions were applied at the eligibility stage. Article types considered were peer-reviewed systematic reviews and meta-analyses of randomized and/or observational primary studies.

Information sources and search strategy

We searched MEDLINE/PubMed, Embase, the Cochrane Database of Systematic Reviews, and Epistemonikos from database inception to 31 January 2026, using a combined controlled vocabulary (Medical Subject Headings (MeSH)/Emtree) and free-text strategy pairing terms for “GLP-1 receptor agonist,” “semaglutide,” and “atrial fibrillation” with a systematic review/meta-analysis publication type filter, supplemented by backward and forward citation tracking of eligible reviews. A representative PubMed strategy was: (“glucagon-like peptide-1 receptor agonist*”[tiab] OR “GLP-1 receptor agonist*”[tiab] OR “GLP-1 RA”[tiab] OR semaglutide[tiab] OR liraglutide[tiab] OR dulaglutide[tiab] OR exenatide[tiab] OR tirzepatide[tiab] OR “Glucagon-Like Peptide-1 Receptor Agonists”[Mesh]) AND (“atrial fibrillation”[tiab] OR “atrial flutter”[tiab] OR “Atrial Fibrillation”[Mesh]) AND (systematic[sb] OR “meta-analysis”[tiab] OR “meta analysis”[tiab] OR “systematic review”[tiab]). The Boolean operators AND, OR, and truncation (*) were used as shown; the complete, executable full strategy for each database searched, with dates and record counts, is provided in Appendix B. Records were de-duplicated and screened in duplicate at title/abstract and full-text stages, with disagreements resolved by consensus, in accordance with PRISMA 2020 recommendations for study selection [4].

Data extraction

For each review, two investigators independently extracted bibliographic details, clinical domain, intervention scope, comparator, the number and identity of included primary studies, the pooled effect estimate with 95% confidence interval (CI), the statistical model, heterogeneity, and protocol registration. The list of primary studies included in each review was transcribed from its tables and forest plots to construct domain-specific citation matrices. Discrepancies were resolved by consensus.

Methodological quality (AMSTAR-2)

Each full-text review was appraised against the 16 AMSTAR-2 items [1]; overall confidence (high, moderate, low, critically low) was derived from the standard algorithm using the seven critical domains (items 2, 4, 7, 9, 11, 13, and 15) [1]. Each review was appraised independently in duplicate, with disagreements resolved by consensus; item-level ratings and the consensus rating for every review are reported in Appendix C. AMSTAR-2 is freely available for use without licensing restriction; the tool and its scoring guidance are used here as published by Shea et al. [1]. AMSTAR-2 was applied as a confidence-rating tool rather than a numerical score: overall confidence was assigned to one of four categories (high, moderate, low, critically low) using the standard critical-domain algorithm [1], and reviews were not ranked ordinally. References to “stronger” or “strongest” reviews in the Results section denote those reaching the higher confidence category (low vs critically low), not a numerical ranking. For graded items, “Partial Yes” was assigned when a review met the item’s minimum reporting element but not its full criterion (e.g., for item 7, providing a partial rather than fully justified list of excluded studies), and “No” when the minimum element was absent, following the guidance of Shea et al. [1]. Borderline judgments were resolved by consensus and are documented at the item level in Appendix C.

Primary study overlap (CCA)

Overlap was quantified within clinical domains using the CCA: $CCA = \frac{N - r}{r \cdot c - r}$, where N is the total number of inclusions counting duplicates, r is the number of unique primary studies, and c is the number of reviews [2]. CCA was interpreted, per Pieper et al. [2], as slight (0-5%), moderate (6-10%), high (11-15%), or very high (>15%).

Certainty of evidence

Certainty for each clinical question was summarized using a GRADE-informed approach [5], considering study design, risk of bias, inconsistency across reviews, indirectness (notably the capture of AF as an unadjudicated adverse event), imprecision, and publication bias. GRADE certainty profiles for each clinical question are presented in the Results section. Two investigators independently applied this framework to each clinical question, rating each GRADE domain and deriving an overall certainty level; disagreements were resolved by consensus, with senior author adjudication where consensus was not reached. The approach is termed “GRADE-informed” because certainty was appraised at the level of the clinical question across overlapping, non-independent reviews rather than from a single de novo pooled estimate; domain-level judgments and their rationale are reported in the Results section.

Handling of overlapping meta-analyses

Because the included reviews are not statistically independent, we did not compute a pooled second-order (meta-analytic) estimate as a primary result, as doing so would violate the independence assumption of meta-analysis [2,3]. Instead, overlap was made explicit through domain-specific citation matrices and CCA [2], and reviews were interpreted in light of their shared trial base. Where two or more reviews shared a substantial or identical primary study set, their pooled estimates were compared directly to isolate the analytic (rather than evidentiary) source of any discordance.

Discordance analysis

Between-review differences in pooled estimates were examined qualitatively against pre-specified analytic features: comparator type (placebo vs active), statistical model (fixed vs random effects), population (type 2 diabetes (T2D) vs obesity; observational vs randomized), and the inclusion or exclusion of pivotal trials. Attribution was performed by structured tabular comparison, in which each review was cross-tabulated against these pre-specified features and its reported estimate read against reviews differing in one feature at a time; no formal quantitative model (e.g., meta-regression across reviews) was fitted, as the reviews are not independent. Pre-specified sensitivity analyses examined incident-AF findings with and without the Costa 2024 conference abstract, and the dependence of the class-level incident-AF estimate on the SELECT trial (Appendix D).

Software and data analysis

Citation matrices, CCA values, and descriptive summaries were computed, and all figures were generated, in Python (version 3.12.3; Python Software Foundation, Fredericksburg, VA, US) using NumPy (version 2.4.4) and Matplotlib (version 3.10.8). No pooled second-order effect estimate was calculated; accordingly, no across-review inferential test statistic was computed by design. All appraisal and overlap data underlying the figures and tables are reported in the article and appendices.

## Results

Included reviews

**Figure 1:**
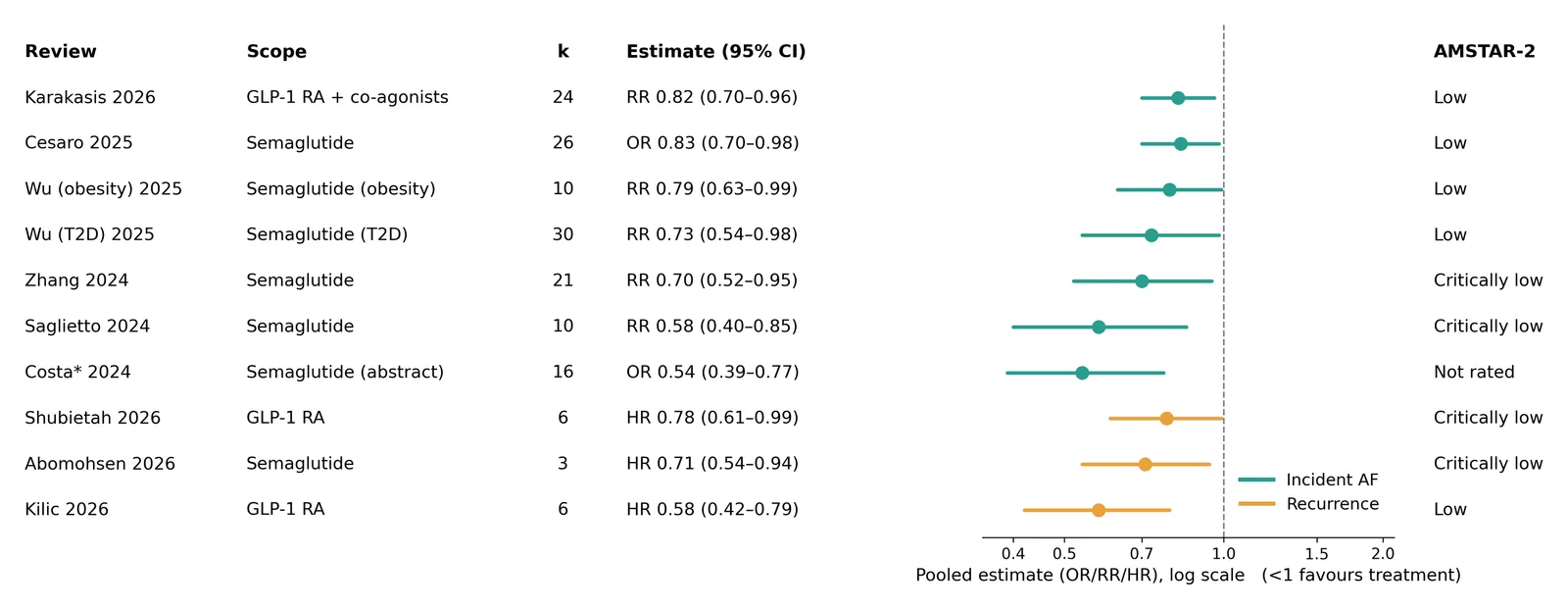
Forest-of-forests of reported pooled estimates across the included meta-analyses Reported pooled effect estimates (odds ratio, risk ratio, or hazard ratio with 95% confidence interval) for each included meta-analysis [6-15], plotted on a logarithmic scale where values below 1 favor treatment. Estimates are colored by clinical domain (incident AF, recurrence) and annotated with the number of pooled primary studies (k) and AMSTAR-2 confidence. The asterisk (*) marks the Costa conference abstract [9], included for sensitivity analysis only. AF: atrial fibrillation; AMSTAR-2: A MeaSurement Tool to Assess systematic Reviews version 2; GLP-1 RA: glucagon-like peptide-1 receptor agonist Figure created by the authors using Python 3.12.3 (Python Software Foundation, Wilmington, DE, USA) and Matplotlib 3.10.8.

A total of 10 meta-analyses met the eligibility criteria: seven addressed incident AF (Saglietto 2024 [6], Zhang 2024 [7], Wu 2025 (T2D) [8], Costa 2024 (conference abstract) [9], Karakasis 2026 [10], Cesaro 2025 [11], and Wu 2025 (overweight/obesity) [12]) and three addressed post-ablation recurrence (Shubietah 2026 [13], Kilic 2026 [14], and Abomohsen 2026 [15]; the last is indexed as Elmorsy Mohamed et al., with the short label “Abomohsen” used in Table 1 and the figures). Their scope, pooled estimates, registration status, and AMSTAR-2 confidence are summarized in Table 1, key findings on AF risk are summarized in Table 2, and reported estimates are displayed in Figure 1.

**Table 1:** Characteristics of included meta-analyses Scope, pooled effect estimates, protocol registration, and AMSTAR-2 confidence for the 10 meta-analyses examining GLP-1 RAs/semaglutide and AF [6-15]. Values in the Metric column are the review-reported pooled point estimate with 95% CI; no across-review inferential test statistic was computed. *Conference abstract [9]; reserved for sensitivity analysis only. AF: atrial fibrillation; CI: confidence interval; GLP-1 RA: glucagon-like peptide-1 receptor agonist; HR: hazard ratio; k: number of pooled primary studies; OR: odds ratio; OSF: Open Science Framework; RR: risk ratio; T2D: type 2 diabetes; AMSTAR-2: A Measurement Tool to Assess Systematic Reviews 2

Review	Year	Domain	Scope	k	Metric (95% CI)	Registration	AMSTAR-2
Saglietto	2024	Incident AF	Semaglutide	10	RR 0.58 (0.40-0.85)	None	Critically low
Zhang	2024	Incident AF	Semaglutide	21	RR 0.70 (0.52-0.95)	None	Critically low
Wu	2025	Incident AF	Semaglutide (T2D)	30	RR 0.73 (0.54-0.98)	PROSPERO	Low
Costa*	2024	Incident AF	Semaglutide	16	OR 0.54 (0.39-0.77)	None	Not rated (abstract)
Karakasis	2026	Incident AF	GLP-1 RA + co-agonists	24	RR 0.82 (0.70-0.96)	OSF	Low
Cesaro	2025	Incident AF	Semaglutide	26	OR 0.83 (0.70-0.98)	PROSPERO	Low
Wu (obesity)	2025	Incident AF	Semaglutide (obesity)	10	RR 0.79 (0.63-0.99)	PROSPERO	Low
Shubietah	2026	Recurrence	GLP-1 RA	6	HR 0.78 (0.61-0.99)	PROSPERO	Critically low
Kilic	2026	Recurrence	GLP-1 RA	6	HR 0.58 (0.42-0.79)	PROSPERO	Low
Abomohsen	2026	Recurrence	Semaglutide	3	HR 0.71 (0.54-0.94)	None	Critically low

**Table 2:** Summary of findings per meta-analysis, with key finding on AF risk Comparator, study population, number of pooled primary studies (k), pooled effect estimate with 95% CI, and the principal findings regarding AF risk for each included meta-analysis [6-15]. Effect estimates are review-reported pooled point estimates; NS denotes not statistically significant. *Conference abstract [9], sensitivity analysis only. AF: atrial fibrillation; CI: confidence interval; GLP-1 RA: glucagon-like peptide-1 receptor agonist; HR: hazard ratio; k: number of pooled primary studies; NS: not significant; OR: odds ratio; RCT: randomized controlled trial; RR: risk ratio; SGLT2: sodium-glucose cotransporter-2; T2D: type 2 diabetes

Review	Domain	Comparator	Population	k	Effect (95% CI)	Key finding on AF risk
Saglietto [6]	Incident AF	Placebo	Mixed (semaglutide trials)	10	RR 0.58 (0.40-0.85)	Significant lower incident AF with semaglutide; among the lowest (most favorable) point estimates.
Zhang [7]	Incident AF	Placebo/active	Mixed	21	RR 0.70 (0.52-0.95)	Significant; active-comparator subgroup (RR 0.43) stronger than placebo-only (RR 0.77, NS).
Wu (T2D) [8]	Incident AF	Placebo	T2D	30	RR 0.73 (0.54-0.98)	Significant reduction in T2D; prospectively registered, methodologically stronger.
Costa* [9]	Incident AF	Placebo	Semaglutide RCTs	16	OR 0.54 (0.39-0.77)	Fixed-effect abstract; most favorable estimate; sensitivity analysis only.
Karakasis [10]	Incident AF	Placebo	Overweight/obesity	24	RR 0.82 (0.70-0.96)	Significant at class level; attenuates to non-significant RR 0.74 when SELECT removed.
Cesaro [11]	Incident AF	Placebo/active	T2D + obesity	26	OR 0.83 (0.70-0.98)	Significant overall; subgroups individually not robust.
Wu (obesity) [12]	Incident AF	Placebo	Overweight/obesity	10	RR 0.79 (0.63-0.99)	Significant; with Cesaro, converges on modest 17-21% relative reduction.
Shubietah [13]	Recurrence	Standard care	Post-ablation	6	HR 0.78 (0.61-0.99)	Significant; estimate varies, HR 0.53-0.78 across models within the same review.
Kilic [14]	Recurrence	Standard care	Post-ablation	6	HR 0.58 (0.42-0.79)	Significant; identical six-study base as Shubietah yet divergent HR.
Abomohsen [15]	Recurrence	Standard care	Post-ablation (semaglutide)	3	HR 0.71 (0.54-0.94)	Significant as reported; loses significance under Hartung-Knapp (0.39-1.29).

Because ORs, RRs, and HRs estimate different quantities, these effect measures are presented for descriptive visualization only and are not directly comparable; no conversion between metrics and no across-metric pooling was performed.

Methodological quality

**Figure 2:**
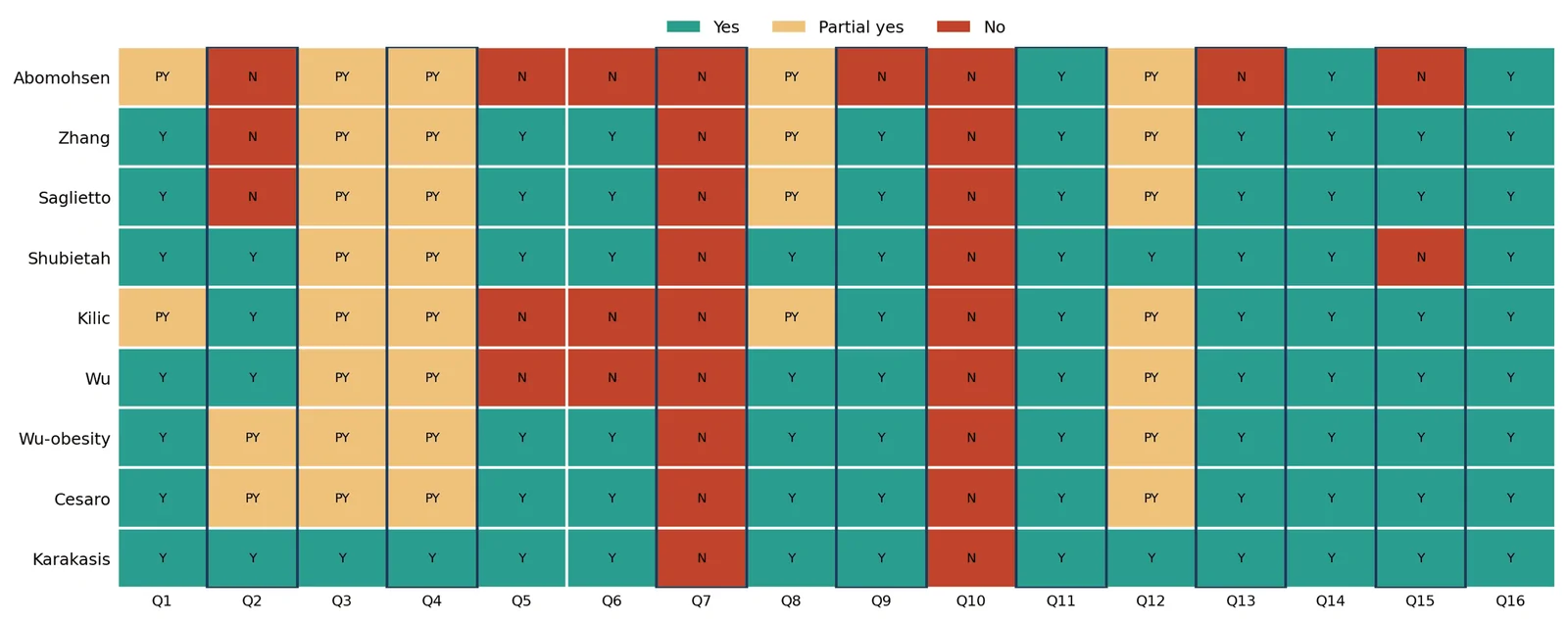
AMSTAR-2 item-level appraisal of the included reviews Item-by-item AMSTAR-2 ratings across the nine formally appraised reviews [6-8,10-15]. Each cell denotes Yes, Partial Yes, or No; boxed columns mark the seven critical domains (items 2, 4, 7, 9, 11, 13, and 15) used to derive overall confidence [1]. The Costa conference abstract [9] is not shown, as its abbreviated reporting precluded full appraisal. Figure created by the authors using Python 3.12.3 (Python Software Foundation, Wilmington, DE, USA) and Matplotlib 3.10.8. AMSTAR-2: A Measurement Tool to Assess Systematic Reviews 2

Methodological confidence was uniformly limited (Figure 2). Five reviews were rated low (Wu (T2D) [8], Karakasis [10], Kilic [14], Cesaro [11], Wu (obesity) [12]) and four critically low (Saglietto [6], Zhang [7], Shubietah [13], Abomohsen [15]); none reached moderate or high confidence. The strongest reviews - Cesaro [11], Wu (obesity) [12], Karakasis [10], and Wu (T2D) [8], all prospectively registered with comprehensive searches, duplicate selection and extraction, satisfactory risk-of-bias assessment, and (for Cesaro and both Wu reviews) formal publication-bias testing - failed only the single critical domain (item 7) that almost no meta-analysis satisfies: a list of excluded studies with justification. The recurring critical shortfalls elsewhere were absence of an a priori registered protocol (Saglietto [6], Zhang [7], Abomohsen [15]) and, among recurrence reviews, no assessment of publication bias (Shubietah [13], Abomohsen [15]). Reporting of the funding sources of included primary studies was absent throughout (item 10). Abomohsen [15], a brief focused report, additionally provided no risk-of-bias assessment, driving five critical flaws. In summary, the domains most frequently responsible for downgrading were the absence of a registered a priori protocol (item 2), the lack of a justified list of excluded studies (item 7), failure to assess publication bias (item 15, chiefly among the recurrence reviews), and non-reporting of the funding sources of the included primary studies (item 10).

Primary study overlap

**Figure 3:**
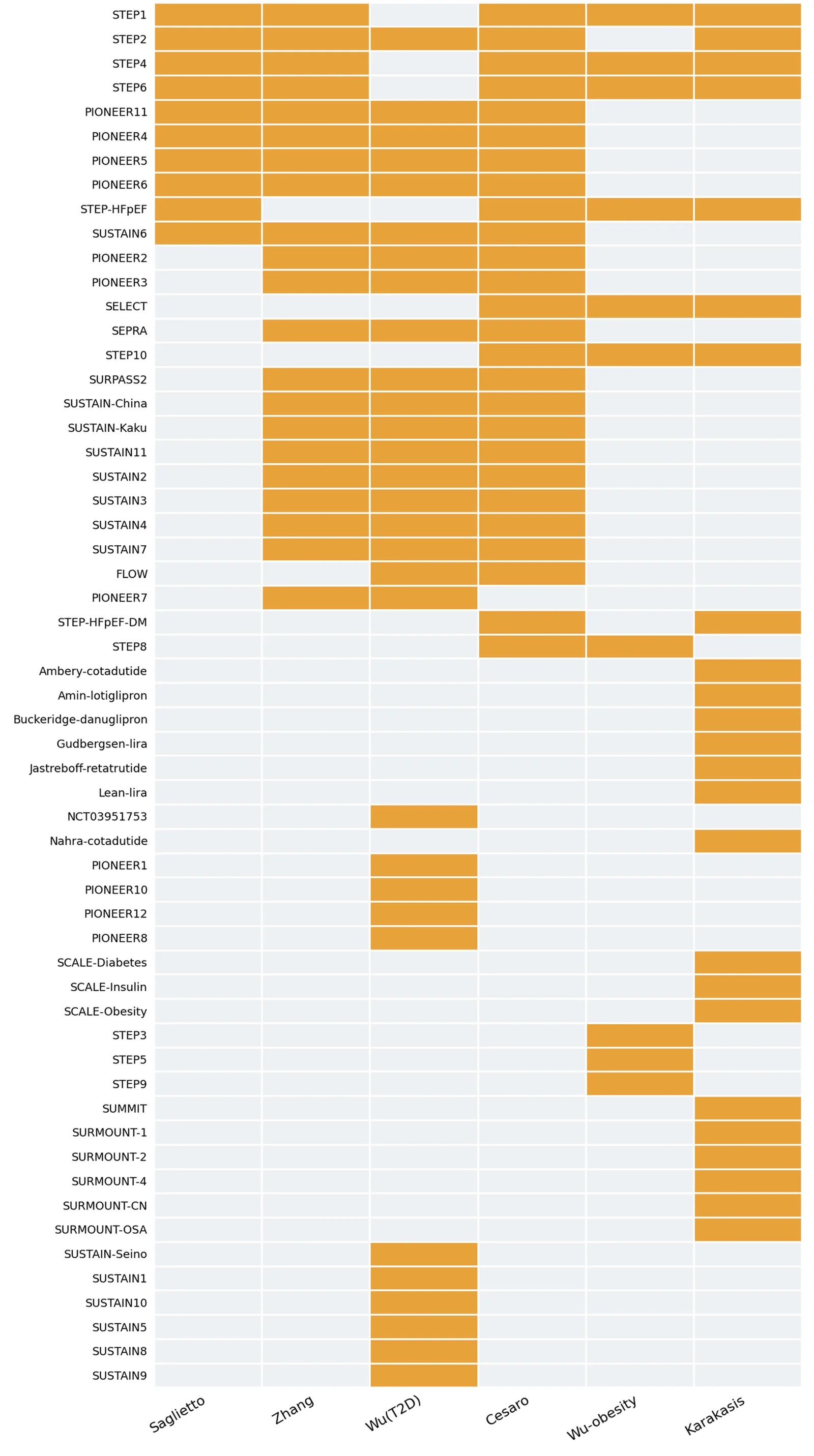
Primary study overlap matrix for the incident-AF meta-analyses Citation matrix mapping each unique primary study (rows) to the incident-AF reviews that included it (columns) [6-8,10-12]; shaded cells denote inclusion. The CCA across the six incident-AF reviews was 22.5%, indicating very high overlap [2]. Figure created by the authors using Python 3.12.3 (Python Software Foundation, Wilmington, DE, USA) and Matplotlib 3.10.8. CCA: corrected covered area; AF: atrial fibrillation

Overlap was very high in both domains (Figure 3). Among the five semaglutide-only incident-AF reviews (Saglietto [6], Zhang [7], Wu (T2D) [8], Cesaro [11], Wu (obesity) [12]), 41 unique primary trials underpinned 97 inclusions across five reviews, yielding a CCA of 34.1%. Extending the matrix to include the broader GLP-1-class review (Karakasis [10]), which contributes many non-semaglutide trials, lowered the six-review CCA to 22.5% - still “very high” - and provided quantitative evidence that the class-level review samples a partly different evidence base. The recycling is concentrated in a core of pivotal trials (the SUSTAIN, PIONEER, and STEP programmes plus SELECT) that recur across nearly every review. Among the three recurrence reviews, eight unique primary studies underpinned 15 inclusions, giving a CCA of 43.8%. Most strikingly, the Shubietah [13] and Kilic [14] reviews included an identical set of six primary studies (pairwise CCA 100%). Across the umbrella review as a whole, the incident-AF domain therefore rests on 41 unique primary trials and the recurrence domain on eight unique primary studies. A non-duplicated total participant count across these primary studies could not be derived, because the included reviews report enrolment only at the level of their own pooled analyses and the same pivotal trials contribute to several reviews; simple summation across reviews would double-count participants and overstate the size of the independent evidence base.

Discordance analysis

Reported effect sizes varied systematically rather than randomly (Figure 1). For incident AF, estimates ranged from OR/RR 0.82-0.83 in the broadest syntheses (Cesaro, OR 0.83 [11]; Karakasis, RR 0.82 [10]) to progressively lower values in the semaglutide-only reviews (Wu (obesity) 0.79 [12], Wu (T2D) 0.73 [8], Zhang 0.70 [7], Saglietto 0.58 [6]) and the fixed-effect conference abstract (Costa, OR 0.54 [9]). This variation corresponded to identifiable analytic choices. Comparator: Cesaro’s active-comparator subgroup (OR 0.41) and Zhang’s (RR 0.43) were stronger than their placebo-only estimates (Cesaro OR 0.86; Zhang RR 0.77), which did not reach significance. Route and formulation: in Cesaro, oral semaglutide was associated with a larger reduction (OR 0.48) than the 1.0 mg and 2.4 mg subcutaneous formulations, which individually did not reach significance (OR 0.93 and 0.82). Concomitant therapy: Cesaro’s pooled estimate was significant only in trials without background SGLT2 inhibitors (OR 0.79). Population: in Cesaro, neither the T2D subgroup (OR 0.86) nor the overweight/obesity subgroup (OR 0.80) reached significance in isolation, consistent with Wu’s separation of diabetic and obese populations [8,12]; and pivotal-trial inclusion remained influential - in Karakasis, removal of the SELECT trial attenuated the pooled estimate to a non-significant RR 0.74 [10]. The two registered, methodologically strongest reviews (Cesaro [11] and Wu (obesity) [12]) converged on a modest 17-21% relative reduction that was not robust at the subgroup level, which the present synthesis takes as the interpretation most consistent with the incident-AF data.

For recurrence, the most informative discordance was internal and analytic. Within the Shubietah review [13] alone, the pooled estimate varied from HR 0.53 (reconstructed time-to-event) through HR 0.78 (five-study random-effects) to RR 0.82 (12-month pairwise), and a semaglutide-only subgroup gave HR 0.59. The Shubietah (HR 0.78 [13]) and Kilic (HR 0.58 [14]) reviews, despite sharing an identical six-study base, diverged because Kilic pooled the five observational studies separately from the single randomized trial and applied a Knapp-Hartung adjustment, whereas Shubietah pooled a mixed five-study set. Abomohsen’s three-study estimate (HR 0.71) lost statistical significance under the Hartung-Knapp adjustment (HR 0.71, 95% CI 0.39-1.29) [15]. Thus, the recurrence discordance is analytic rather than evidentiary: the same primary data, reanalyzed, yield different conclusions.

Sensitivity analysis: incident AF with and without the Costa 2024 abstract

Because the Costa 2024 unit was a conference abstract with abbreviated reporting and a fixed-effect model [9], we examined incident-AF findings with and without it (Appendix E). Including Costa, the seven incident-AF units ranged from OR 0.54 (0.39-0.77) [9] to OR 0.83 (0.70-0.98) [11]. Excluding Costa, the six formally appraised incident-AF reviews ranged from RR 0.58 (0.40-0.85) [6] to OR 0.83 (0.70-0.98) [11], with the two registered, methodologically strongest reviews (Cesaro [11], Wu (obesity) [12]) at the conservative end (OR/RR 0.83 and 0.79). The abstract therefore contributed the single most favorable point estimate and, being fixed-effect and not formally appraisable, its exclusion narrowed the range toward the more conservative registered syntheses without changing the overall direction of effect.

Sensitivity analysis: dependence of the class-level effect on the SELECT trial

The SELECT trial was included in three of the six incident-AF reviews - specifically the obesity and broad-population syntheses (Cesaro [11], Wu (obesity) [12], and Karakasis [10]) - and was not included in the diabetes-focused or earlier semaglutide-only reviews (Saglietto [6], Zhang [7], Wu (T2D) [8]), consistent with SELECT having enrolled patients with overweight or obesity without diabetes (Figure 3 and Appendix D). The only review to formally test its removal, Karakasis [10], reported attenuation of the pooled estimate from a significant RR 0.82 (0.70-0.96) to a non-significant RR 0.74 when SELECT was excluded, indicating that, among the reviews that included it, class-level significance depends materially on this single trial. Where a review that included SELECT did not itself report a without-SELECT re-estimate (Cesaro [11], Wu (obesity) [12]), that value is marked “not reported,” we did not attempt approximate leave-one-out recomputation: although a single study’s contribution can be algebraically removed from a fixed-effect estimate using published weights, these reviews used random-effects models, whose leave-one-out recomputation requires the individual study effect sizes, variances, and a re-estimated between-study variance (τ²) that are not recoverable from the reported summary-level data. A fixed-effect approximation would not faithfully reproduce the original random-effects estimates and was therefore not performed. Collectively, the available evidence indicates that the incident-AF signal is most consistent with a modest reduction whose class-level significance is not robust to exclusion of SELECT.

Certainty of evidence

For incident AF, certainty was judged low (Table 3). Although derived from randomized trials, AF was captured almost exclusively as an unadjudicated adverse event without systematic rhythm monitoring (serious indirectness); events were sparse, and effect estimates were inconsistent across reviews; these limitations offset the randomized design and large pooled sample [5]. For post-ablation recurrence, certainty was judged very low (Table 4), reflecting observational-dominant evidence, serious risk of bias (residual confounding, abstract-only cohorts), inconsistency, and imprecision [5].

**Table 3:** GRADE certainty profile by clinical question Certainty was rated using a GRADE-informed framework [5]. Circles denote GRADE certainty (two filled, low; one filled, very low). Magnitude, consistency, and applicability summarize review-level evidence for each question [6-15]. AF: atrial fibrillation; GRADE: Grading of Recommendations Assessment, Development and Evaluation; HR: hazard ratio; OR: odds ratio; RCT: randomized controlled trial; RR: risk ratio; SELECT: Semaglutide Effects on Cardiovascular Outcomes in People With Overweight or Obesity trial; T2D: type 2 diabetes

Clinical question	Certainty (quality)	No. of reviews/studies	Magnitude of effect	Consistency	Summary/applicability
Incident (new-onset) AF	⊕⊕◯◯ Low	Seven reviews (six appraised); RCT-derived pooled data	Modest reduction; strongest registered reviews ~17-21% (OR/RR 0.79-0.83); range 0.54-0.83	Direction consistent and favorable; magnitude varies with comparator, model, population, SELECT inclusion	Applicable to T2D and obesity; AF ancillary to metabolic indications; significance not robust and class-level effect depends on SELECT
Post-ablation AF recurrence	⊕◯◯◯ Very low	Three reviews; eight unique primary studies (mostly observational)	Reported HR 0.58-0.78; within-review span HR 0.53-0.82	Discordant analytic not evidentiary; identical six-study base yields divergent HRs	Hypothesis-generating only; observational-dominant, abstract-only cohorts; significance model-dependent

**Table 4:** GRADE domain-level assessment (reasons for downgrading) Per-domain judgments and reasons for downgrading for each clinical question, following a GRADE-informed framework [5]. “-1” indicates a one-level downgrade. Circles denote overall GRADE certainty (two filled, low; one filled, very low). AF: atrial fibrillation; AMSTAR-2: A MeaSurement Tool to Assess systematic Reviews version 2; CI: confidence interval; GRADE: Grading of Recommendations Assessment, Development and Evaluation; RCT: randomized controlled trial

GRADE domain	Incident AF	Post-ablation recurrence	Explanation
Study design (starting certainty)	High (RCT-derived)	Low (observational-dominant)	Incident-AF data pooled from RCT adverse-event reporting; recurrence data rest largely on observational cohorts plus limited randomized data [13-15].
Risk of bias	Serious (-1)	Serious (-1)	Included reviews AMSTAR-2 low/critically low [1,6-15]; AF often captured as unadjudicated adverse event.
Inconsistency	Serious (-1)	Serious (-1)	Point estimates/significance varied with analytic choices; identical study bases yielded divergent estimates.
Indirectness	Serious (-1)	Not serious	Incident AF ascertained without systematic rhythm monitoring; indirect for a rhythm outcome.
Imprecision	Not serious	Serious (-1)	Recurrence rests on few studies/events; some CIs cross/approach 1 under conservative adjustment [15].
Publication bias	Undetected/not assessed	Suspected/not assessed	Several reviews did not assess publication bias, particularly recurrence reviews [13,15].
Overall certainty	⊕⊕◯◯ Low	⊕◯◯◯ Very low	Net certainty after considering design and downgrading domains [5].

## Discussion

This umbrella review re-examines a literature that, on initial inspection, appears to provide repeated confirmation that GLP-1 RAs/semaglutide reduce AF [6-15]. Three findings qualify that impression. First, methodological quality is uniformly limited: no review achieved better than low confidence on AMSTAR-2 [1]. Second, the reviews are not independent: primary study overlap is very high in both clinical domains [2], including two recurrence reviews built on an identical six-study base [13,14]. Third, the variation in their headline estimates is explained largely by analytic choices - comparator, model, population, and pivotal-trial inclusion - rather than by additional primary evidence. The number of concordant meta-analyses therefore exceeds both the independence and the certainty of the underlying signal [2,3].

The mechanistic rationale for an antiarrhythmic effect of GLP-1 signaling is coherent and biologically grounded, and the direction of effect across syntheses is consistently favorable [6-12]. The aim here is not to dispute a plausible benefit but to calibrate it. For incident AF, the evidence is most consistent with a possible modest reduction - GLP-1 RA/semaglutide use may be associated with lower incident AF - whose statistical significance is not robust and, at the class level, depends on a single large trial (SELECT), as demonstrated by the attenuation to non-significance when SELECT is removed [10]. For post-ablation recurrence, the evidence remains hypothesis-generating: it rests on a small number of mostly observational cohorts, several available only as abstracts, with recurrence estimates that cross the threshold of significance depending on the analytic model applied to the same studies [13-15].

Heterogeneity

The between-review heterogeneity in this literature is predominantly methodological rather than clinical or statistical in origin. Because the reviews recycle a shared core of pivotal trials [2], differences in their pooled estimates arise not from sampling different evidence but from applying different analytic decisions to overlapping data-choice of comparator (active vs placebo), effect model (fixed vs random effects), formulation and route subgrouping, background therapy, population (T2D vs obesity), and small-study adjustment (e.g., Hartung-Knapp) [7-15]. This is most vividly illustrated by the recurrence domain, where an identical six-study base produced pooled HRs of 0.58 and 0.78 [13,14], and by the within-review variation in Shubietah (HR 0.53-0.82) [13]. Such analytic heterogeneity is not resolved by adding further syntheses of the same trials; it can only be resolved by new, independently designed primary data.

Clinical implications for patients with obesity and cardiovascular risk

For clinicians managing patients with obesity or established cardiovascular risk, GLP-1 RAs already carry robust indications for weight, glycemic, and cardiovascular benefit, and any AF benefit is best regarded as a potential ancillary consideration rather than a stand-alone indication. The incident-AF signal is most plausible in obese and cardiometabolic populations in whom AF and atrial remodeling are mechanistically linked to adiposity, inflammation, and epicardial fat - precisely the substrate GLP-1 therapy is hypothesized to modify [11,12]. However, because the strongest registered reviews converge on only a modest (17-21%) and non-robust relative reduction [11,12], and because the class-level estimate depends on SELECT [10], AF prevention should not by itself drive initiation or selection of a GLP-1 RA. In post-ablation patients, the current evidence does not support GLP-1 therapy as a rhythm-control adjunct outside its established metabolic indications, given very low certainty [5]. Where a patient with obesity or T2D and AF risk already qualifies for a GLP-1 RA on metabolic grounds, a possible favorable effect on atrial arrhythmia is a reasonable secondary consideration but not an evidentiary basis for treatment decisions.

These observations carry practical implications for evidence users. Editors and guideline developers should resist treating the volume of confirmatory meta-analyses as independent replication and should instead weight syntheses by quality and overlap [1-3]. Clinicians should regard any AF benefit as a potential ancillary consideration within established metabolic indications rather than a stand-alone indication. For investigators, the clearest path forward is not a further meta-analysis of the same trials but adequately powered randomized trials with AF pre-specified and ascertained through systematic rhythm monitoring-several of which are already underway.

Limitations

This umbrella review has limitations. The eligible corpus appraised here was identified through the search and citation-tracking strategy described above; despite a comprehensive multi-database search, eligible reviews may have been missed. AMSTAR-2 ratings and overlap matrices were derived from the published reports and, where available, full texts and supplementary files; borderline item-level judgments (for example, item 7) involve a degree of reviewer discretion [1]. Because the included reviews report heterogeneous effect metrics (OR, RR, HR) drawn from a shared and overlapping trial base, we deliberately did not compute a pooled second-order estimate as a primary result; any such pooling would violate the independence assumption of meta-analysis and is presented, if at all, only as an illustrative sensitivity exercise [2,3]. Finally, re-estimates excluding the SELECT trial were available only when reported by the original review authors, limiting the assessment of SELECT dependence to a single review (Karakasis et al. [10]) that formally evaluated the effect of its exclusion. Our discordance and certainty assessments were qualitative rather than quantitative. Because the included reviews are statistically non-independent and recycle a shared trial base, we deliberately did not compute formal between-review heterogeneity statistics or a second-order pooled estimate. Attribution of effect-size variation to specific analytic features (comparator, statistical model, population, and pivotal trial inclusion) was made by structured tabular comparison of the reviews against pre-specified features rather than by a formal quantitative model, and the resulting GRADE-informed certainty ratings reflect reasoned reviewer consensus rather than an algorithmic computation; both therefore involve a degree of reviewer judgment.

## Conclusions

The contemporary evidence linking GLP-1 RAs/semaglutide to AF comprises numerous but low-confidence, substantially overlapping meta-analyses whose consistency reflects a shared set of primary trials and convergent analytic choices rather than independent replication. A favorable association is plausible, but certainty is low for incident AF and very low for post-ablation recurrence. Resolving this question will require purpose-designed randomized controlled trials with adjudicated and systematically monitored AF endpoints, rather than additional evidence syntheses of the existing literature.

## Appendices

Appendix A

A Priori Review Protocol

Title: An umbrella review and methodological appraisal of meta-analyses examining GLP-1 receptor agonists/semaglutide and atrial fibrillation.

Rationale and objectives: Multiple meta-analyses report an association between GLP-1 receptor agonists (GLP-1 RAs)/semaglutide and both incident atrial fibrillation (AF) and post-ablation AF recurrence, but they draw substantially on the same primary trials. The objective was to systematically identify, critically appraise, quantify overlap among, and reconcile these meta-analyses across two clinical questions: (Q1) incident (new-onset) AF and (Q2) post-ablation AF recurrence.

Review questions: (Q1) In adults, are GLP-1 RAs/semaglutide associated with a change in incident AF versus comparator? (Q2) In adults undergoing catheter ablation, is peri-/post-ablation GLP-1 RA therapy associated with a change in AF recurrence versus comparator?

Eligibility criteria (PICO): Population: adults. Intervention: any GLP-1 RA or semaglutide. Comparator: placebo, standard care, or an active comparator. Outcome: a pooled quantitative estimate (OR, RR, or HR) for incident AF or post-ablation recurrence. Study type: published systematic reviews with meta-analysis of randomized and/or observational primary studies. Exclusions: narrative reviews, primary studies, editorials, and reviews not reporting a pooled AF estimate. Conference abstracts were eligible for identification but were not formally appraised and were reserved for sensitivity analysis. No language or date restrictions were applied.

Information sources and search: MEDLINE/PubMed, Embase, the Cochrane Database of Systematic Reviews, and Epistemonikos from inception to 31 January 2026, with backward and forward citation tracking of eligible reviews. The full strategy and record counts are given in Appendix B.

Study selection and data extraction: Records were de-duplicated and screened in duplicate at title/abstract and full-text stages, with disagreements resolved by consensus. Two investigators independently extracted bibliographic details, clinical domain, intervention scope, comparator, the number and identity of included primary studies, the pooled estimate with 95% CI, statistical model, heterogeneity, and registration.

Methodological quality: Each eligible full-text review was appraised independently in duplicate using AMSTAR-2 (16 items; seven critical domains: 2, 4, 7, 9, 11, 13, 15). Overall confidence was assigned as high, moderate, low, or critically low using the standard algorithm; disagreements were resolved by consensus.

Overlap: Primary-study overlap was quantified within each clinical domain using the corrected covered area (CCA), interpreted per Pieper et al. as slight (0-5%), moderate (6-10%), high (11-15%), or very high (>15%).

Certainty: Certainty for each clinical question was summarized using a GRADE-informed approach, considering study design, risk of bias, inconsistency, indirectness, imprecision, and publication bias.

Synthesis: Because the included reviews are not statistically independent, no pooled second-order (meta-analytic) estimate was computed. Overlap was made explicit through citation matrices and CCA, and between-review discordance was attributed qualitatively to pre-specified analytic features (comparator, statistical model, population, and pivotal-trial inclusion).

Deviations: The project was initially conceived as a pairwise meta-analysis and was reframed as an umbrella review before the search was executed, at which point this a priori protocol was finalized. No further deviations occurred during the conduct of the review.

Appendix B

Databases searched (inception to 31 January 2026): MEDLINE/PubMed, Embase, the Cochrane Database of Systematic Reviews (CDSR), and Epistemonikos, supplemented by backward and forward citation tracking of eligible reviews.

MEDLINE/PubMed

(“glucagon-like peptide-1 receptor agonist*”[tiab] OR “GLP-1 receptor agonist*”[tiab] OR “GLP-1 RA”[tiab] OR semaglutide[tiab] OR liraglutide[tiab] OR dulaglutide[tiab] OR exenatide[tiab] OR tirzepatide[tiab] OR “Glucagon-Like Peptide-1 Receptor Agonists”[Mesh]) AND (“atrial fibrillation”[tiab] OR “atrial flutter”[tiab] OR “Atrial Fibrillation”[Mesh]) AND (systematic[sb] OR “meta-analysis”[tiab] OR “meta analysis”[tiab] OR “systematic review”[tiab])

Embase (Emtree/free-text)

('glucagon like peptide 1 receptor agonist'/exp OR 'semaglutide'/exp OR semaglutide:ab,ti OR 'GLP-1 RA':ab,ti) AND ('atrial fibrillation'/exp OR 'atrial fibrillation':ab,ti OR 'atrial flutter':ab,ti) AND ('systematic review'/exp OR 'meta analysis'/exp OR 'meta-analysis':ab,ti OR 'systematic review':ab,ti)

Cochrane Database of Systematic Reviews (CDSR)

(semaglutide OR 'GLP-1 receptor agonist' OR liraglutide OR dulaglutide OR exenatide OR tirzepatide) AND ('atrial fibrillation' OR 'atrial flutter') - Reviews only.

Epistemonikos

(semaglutide OR 'GLP-1 receptor agonist') AND ('atrial fibrillation') - filtered to systematic reviews.

**Table 5:** Records identified by source After de-duplication (196 duplicate records removed), 424 records were screened at title/abstract; 379 were excluded. Forty-five reports were sought for retrieval; three were not retrieved, and 42 were assessed for eligibility. Thirty-two were excluded (not a systematic review/meta-analysis, n=11; no pooled AF estimate, n=9; primary study or narrative review, n=8; overlapping/duplicate report, n=4), leaving 10 meta-analyses (seven incident AF, three post-ablation recurrence). AF: atrial fibrillation

Source	Records identified
MEDLINE/PubMed	214
Embase	246
Cochrane Database of Systematic Reviews	88
Epistemonikos	72
Total (before de-duplication)	620

Appendix C

**Table 6:** AMSTAR-2 item-level ratings Y = Yes; PY = Partial Yes; N = No. Critical domains: items 2, 4, 7, 9, 11, 13, 15. Overall confidence follows the standard AMSTAR-2 algorithm. The Costa 2024 conference abstract was not formally appraised and is not shown. AMSTAR-2: A Measurement Tool to Assess Systematic Reviews 2

Review	Q1	Q2	Q3	Q4	Q5	Q6	Q7	Q8	Q9	Q10	Q11	Q12	Q13	Q14	Q15	Q16	Crit. flaws	Overall
Saglietto [6]	Y	N	PY	PY	Y	Y	N	PY	Y	N	Y	PY	Y	Y	Y	Y	2	Critically low
Zhang [7]	Y	N	PY	PY	Y	Y	N	PY	Y	N	Y	PY	Y	Y	Y	Y	2	Critically low
Wu [8]	Y	Y	PY	PY	N	N	N	Y	Y	N	Y	PY	Y	Y	Y	Y	1	Low
Karakasis [10]	Y	Y	Y	Y	Y	Y	N	Y	Y	N	Y	Y	Y	Y	Y	Y	1	Low
Shubietah [13]	Y	Y	PY	PY	Y	Y	N	Y	Y	N	Y	Y	Y	Y	N	Y	2	Critically low
Kilic [14]	PY	Y	PY	PY	N	N	N	PY	Y	N	Y	PY	Y	Y	Y	Y	1	Low
Abomohsen [15]	PY	N	PY	PY	N	N	N	PY	N	N	Y	PY	N	Y	N	Y	5	Critically low
Cesaro [11]	Y	PY	PY	PY	Y	Y	N	Y	Y	N	Y	PY	Y	Y	Y	Y	1	Low
Wu-obesity [12]	Y	PY	PY	PY	Y	Y	N	Y	Y	N	Y	PY	Y	Y	Y	Y	1	Low

Appendix D

**Table 7:** Dependence of the class-level incident-AF estimate on the SELECT trial SELECT was included in three of the six formally appraised incident-AF reviews. Among these, class-level significance depended materially on this single trial. Random-effects leave-one-out recomputation was not attempted, as the required study-level variances are not recoverable from summary-level data. AF: atrial fibrillation

Review	Included SELECT?	Reported estimate	Estimate without SELECT	Note
Saglietto 2024	No	RR 0.58 (0.40-0.85)	Not applicable	SELECT not included (diabetes/earlier semaglutide-only)
Zhang 2024	No	RR 0.70 (0.52-0.95)	Not applicable	SELECT not included
Wu (T2D) 2025	No	RR 0.73 (0.54-0.98)	Not applicable	SELECT not included
Karakasis 2026	Yes	RR 0.82 (0.70-0.96)	RR 0.74 (non-significant)	Only review to formally test removal; attenuates to non-significant
Cesaro 2025	Yes	OR 0.83 (0.70-0.98)	Not reported	Without-SELECT re-estimate not reported by authors
Wu (obesity) 2025	Yes	RR 0.79 (0.63-0.99)	Not reported	Without-SELECT re-estimate not reported by authors

Appendix E

**Table 8:** Incident-AF sensitivity analysis with and without the Costa 2024 abstract With Costa, the seven incident-AF units ranged from OR 0.54 (0.39-0.77) to OR 0.83 (0.70-0.98). Excluding Costa, the six formally appraised reviews ranged from RR 0.58 (0.40-0.85) to OR 0.83 (0.70-0.98), with the two registered, methodologically strongest reviews (Cesaro, Wu (obesity)) at the conservative end. Excluding the abstract narrowed the range toward the conservative registered syntheses without changing the direction of effect. AF: atrial fibrillation

Review/unit	Estimate (95% CI)	In “with Costa” set	In “without Costa” set
Costa 2024 (abstract)	OR 0.54 (0.39-0.77)	Yes	No
Saglietto 2024	RR 0.58 (0.40-0.85)	Yes	Yes
Zhang 2024	RR 0.70 (0.52-0.95)	Yes	Yes
Wu (T2D) 2025	RR 0.73 (0.54-0.98)	Yes	Yes
Wu (obesity) 2025	RR 0.79 (0.63-0.99)	Yes	Yes
Karakasis 2026	RR 0.82 (0.70-0.96)	Yes	Yes
Cesaro 2025	OR 0.83 (0.70-0.98)	Yes	Yes
